# Delivery of interpreting services in UK primary care by population needs: a multisite case study

**DOI:** 10.1136/bmjph-2025-003454

**Published:** 2026-03-31

**Authors:** Judith Yargawa, Cecilia Vindrola-Padros, Katriina L Whitaker, Graham Hieke, Paramjit Gill, Emily D Williams, Lily Islam, Sabine Braun, Georgia B Black

**Affiliations:** 1 Wolfson Institute of Population Health, Queen Mary University of London, London, UK; 2 Department of Targeted Intervention, University College London, London, UK; 3 School of Health Sciences, University of Surrey, Guildford, UK; 4 Warwick Applied Health, Warwick Medical School, University of Warwick, Coventry, UK; 5 Faculty of Life Sciences and Medicine, King’s College London, London, UK; 6 Patient and Public Involvement, London, UK; 7 Centre for Translation Studies, University of Surrey, Guildford, UK

**Keywords:** Communication, Health Services Accessibility, Public Health, Qualitative Research, Social Medicine

## Abstract

**Introduction:**

Interpreting services for patients with no/limited English proficiency are key in healthcare communication and quality of care; yet little evidence exists on interpreting service delivery. This study investigated delivery of interpreting services in UK primary care, particularly how models differ by population needs, and contextual factors impacting delivery.

**Methods:**

Four primary care sites were purposively selected to represent a range of population and practice characteristics. 24 episodes/spaces were observed and semistructured interviews were conducted with 22 frontline staff. Data were analysed using the Rapid Research and Evaluation Lab (RREAL) Rapid Assessment Procedure sheets and inductive thematic analysis.

**Results:**

General practices typically fell under three categories of interpreting needs (high, moderate and low), with models and modalities of interpreting delivery designed around these differing needs. High needs practices, with high migrant populations, had on-site interpreting due to impracticalities of arranging per-appointment interpreting and the perception that face-to-face interpreting is the gold standard. In contrast, low needs areas primarily used telephone interpreting due to perceived ease of access, variety of languages and interpreters and mitigating interpreter travel constraints in rural areas. Similarities across sites included using Google Translate as a supporting interpreting tool and the rarity of video interpreting. Frontline staff across sites held similar expectations of interpreters (having knowledge, interpersonal skills and social/cultural acumen) and interpreting service providers (smooth information technology, high standards and language variety). Factors impacting interpreting delivery were needs-specific and cross-cutting. High needs sites experienced resource pressures, ‘mop-up’ work from secondary care, patients having complex needs and doing extra ‘hidden’ work such as handling non-medical problems. All sites encountered issues with interpreting delivery systems.

**Conclusions:**

Interpreting services tailored to population needs and primary care priorities are fundamental to delivery of interpreting services. Policy and national guidance should take language need into account and support local contexts to deliver approaches tailored to their needs.

**WHAT IS ALREADY KNOWN ON THIS TOPIC**

- People with limited English proficiency face additional obstacles to accessing primary care compared with those with proficient English, with implications for their health outcomes. Little evidence exists on interpreting service delivery in primary care.

**WHAT THIS STUDY ADDS**

- This is the first study to our knowledge to report on the delivery and implementation of interpreting services in UK primary care. It builds on previous studies to consider wider aspects of interpreting delivery such as planning, booking, technology and staffing.
- Our paper describes how local population needs shape the implementation with intended and unintended consequences. Our study has immediate relevance to all healthcare providers, as it provides a rich, in-depth account of everyday interpreting service use and delivery across a wide range of settings. This makes our findings highly generalisable.
- A particular novelty of our study is the use of contrasting case study sites in areas of high, moderate and low interpreting need.

**HOW THIS STUDY MIGHT AFFECT RESEARCH, PRACTICE OR POLICY**

- The findings contribute key evidence on the importance of tailoring interpreting service delivery efforts to the local context. It underscores the need for primary care providers to have a range of options and support to tailor services to their population needs.

## Introduction

 Demand for primary care services in the UK has grown alongside an increasingly diverse patient population. In 2021, over 1 million people or 9% of the population in England and Wales did not speak English well or at all.^1^ Effective communication is fundamental to healthcare delivery, and language barriers can compromise quality of care, patient safety and health outcomes.^2–4^ Primary care forms the majority of healthcare delivery in the UK’s National Health Service (NHS), with 98% of people registered with a general practitioner (GP)^5^ and over 90% of NHS activity in primary care.^6^ Previous studies have shown that people with limited English proficiency face additional obstacles to accessing primary care compared with those with proficient English,^7^ with health implications.^5,8–11^ This is particularly important in primary care, which relies heavily on communication and relationship-building for patient safety and clinical effectiveness.^12,13^

Interpreting services allow patients with limited English proficiency to be understood and receive quality care, therefore reducing healthcare inequalities and improving health outcomes. However, our previous study suggests that only 63% of people with no/limited English proficiency have accessed professional interpreting in primary care.^14^ A survey of primary care providers in Switzerland identified several barriers to adequate interpreting service delivery, including organisational difficulties, poor financing and insufficient knowledge about arranging interpreters.^15^ Provision of interpreting needs to also account for patient preferences with respect to modality (eg, face-to-face, telephone), characteristics such as gender and dialect,^16^ and opportunities and risks associated with technology-enabled models such as Google Translate.^17–20^

Recent government documents have highlighted the importance of access to interpreting services in reducing health inequalities, ensuring healthcare accessibility and improving population outcomes.^21,22^ There is a significant implementation gap in understanding variation in delivery and implementation of interpreting services in UK primary care settings, particularly with respect to how services are tailored to meet the needs of different population groups. Research suggests that patients in areas with greater language diversity and lower English proficiency may face unique barriers to accessing care, which could be exacerbated by inconsistent service delivery models.^23^

There is currently very little evidence about interpreting service delivery, experiences or implementation in UK primary care, particularly how delivery is tailored to differing levels of language need. Previous studies have focused on refugee or asylum seeker populations,^24–27^ hence interpreting for long-term, multigenerational residents remains widely unknown. This important gap in knowledge could help mitigate language barriers more effectively in policy and practice.

This multisite case study aimed to investigate the delivery and implementation of interpreting services in UK primary care, with a particular focus on how priorities and models of service delivery differ by population needs. Specifically, the study aimed to:

- **Compare the delivery of interpreting services** across primary care case study sites located in areas of varying levels of language need.
- **Identify contextual factors** that impact delivery in different local contexts.

## Materials and methods

### Theoretical framework

We used a multisite case study approach underpinned by the Consolidated Framework for Implementation Research (CFIR).^28,29^ The CFIR includes five main constructs that were used to structure our data collection and analysis:

- **Intervention characteristics**: Design, complexity and quality of interpreting delivery.
- **Outer setting**: National policies, local policy, guidance, contracting and commissioning procedures.
- **Inner setting**:
  - Practice characteristics, climate, organisational structure, technology.
  - Interpreting provider characteristics, operational structure.
- **Characteristics of individuals**: Knowledge and experiences of interpreting delivery, training on interpreting.
- **Process**: Tasks, actions and planning for interpreting delivery.

### Sampling

Four primary care sites were purposively selected to represent a range of population and practice characteristics. Site recruitment was informed by advice from the National Institute for Health and Care Research Delivery Networks (formerly Clinical Research Networks) and our advisory and steering group members. Sites were chosen from four distinct geographical areas across England. A quota sampling approach was used to ensure variation in South Asian languages spoken, local deprivation levels, ethnic density and practice size (online supplemental file 1).

### Data collection

A researcher (JY- experienced female qualitative researcher) conducted visits at each site to observe key events related to interpreting services. These included interpreter booking processes, technology use, consultations (in-person, telephone and hybrid) and documentation (eg, flowcharts providing information to clinicians on accessing interpreting services). A three-part observational approach was followed: (1) familiarisation with staff and interpreting services; (2) targeted observations of specific events; and (3) broad observations to capture everyday practices relating to interpreting and language need. Field notes were taken during and after sessions.

Semi-structured interviews were conducted with primary care staff (GPs, nurses, allied clinicians, receptionists and administrative staff) by JY either face-to-face at the practices or via Microsoft Teams. Interviews explored their experiences of interpreting service delivery and the site’s planning and delivery of interpreting services (online supplemental file 2). All interviews were audio-recorded or video-recorded and transcribed, with field notes taken. Interviews lasted approximately 40–45 minutes on average. Data were collected between September 2023 and May 2024. This study was part of a broader study on interpreting services in UK primary care which included interviews with different groups including patients, interpreters, interpreting service providers, national stakeholders and commissioners.^30^ The data from these interviews are beyond this paper’s scope, but they broadly informed interpretation of findings.

### Analysis

Data were analysed using RREAL Rapid Assessment Procedure (RAP) sheets,^31^ which are a way of collating detailed notes and summaries under relevant categories that can iterate and become more detailed as data collection progresses (online supplemental file 3). We had one RAP sheet per case study site to enable cross-case comparison and triangulation. Inductive thematic analysis was also employed to identify key themes within CFIR domains to generate cross-case findings. Data analysis was iterative, with transcribed interviews and observation notes reviewed during data collection. RAP sheets were used for preliminary analysis and to facilitate group discussion (JY, GBB, CVP, KLW, GH). NVivo and Microsoft Office applications were used to manage the data.

### Patient and public involvement

A patient and public involvement lead from the South Asian community (LI) was involved from onset to completion of the project, including design, identification of fieldwork locations and research participants, development of study tools and data interpretation. Patient voice was embedded within our research, with patient views explored on observing day-to-day interpreting in GP surgeries.

### Reporting

This paper has been written in accordance with the Consolidated Criteria for Reporting Qualitative Research (COREQ) checklist.^32^ Participants’ quotes have been reported in italics and quotation marks.

## Results

### Overview of case study sites, participants and data sources

The four case study sites reflect diversity in terms of England regions (two in Greater London, one in South-east and one in Yorkshire), deprivation level (Index of Multiple Deprivation score range 1–6)^33^ and practice size (6000 to >25 000 patients) (online supplemental file 1). Twenty-two frontline staff participated in interviews (5 or 6 staff per site). Twelve of these were clinicians and 10 administrative staff. Six of the respondents were men and 16 women, from different ethnicities (online supplemental file 4).

Twenty-four episodes/spaces were observed across all sites, including 16 interpreted consultations (7 telephone, 6 face-to-face and 3 hybrid), 3 waiting areas, 2 staff offices, 2 bilingual staff interpreted consultations and 1 patient social group (online supplemental file 5). Four documents were analysed, all of which were either flowcharts, tables or texts providing information to clinicians on accessing interpreting services.

### Levels of interpreting needs

While we recruited case study sites to reflect diversity in terms of demographics and delivery (online supplemental file 1), we found that general practices typically fell under three categories of interpreting needs:

- **High needs**: These practices had a significant proportion of patients needing interpreting, located in areas with high migrant populations and had one or a few predominant languages. Sites A and B of our case study were classified as high needs where frontline staff in Site B, for example, reported that over 70% of their patients did not have English as a first language and nearly two-thirds of consultations required an interpreter. Respondents reported that some of these patients were able to get by with limited English for the local shops and social activities, yet struggled with describing medical terms and symptoms and may not feel confident navigating health-related conversations.
- **Low needs**: These practices had very low proportions of patients needing interpreters. Clinicians experienced prolonged periods without any interpreted consultations, for example, one GP having this once every 6–8 weeks. They were located in less deprived areas and/or places with working age people. Site C in our case study fell into this category.
- **Moderate needs**: These practices had need levels between the high and low needs areas. It is quite difficult to estimate a specific percentage, but around 15–30% of patients would need interpreting. Site D in our case study fell into this category.

### Models and modalities of interpreting delivery

Below, we provide findings on models and modalities of interpreting service delivery by level of population needs (table 1), including rationale on options, models, design and impacts.

**Table 1:** Typology of interpreting models and modalities across sites

Level of interpreting needs of patient pop.	Site # and location	Main South Asian ethnicity in patient pop.	Key models of interpreting	Main modality of interpreting	Number of telephone interpreting services in use
High	Site A (Greater London)	Bangladeshi	Health advocate (in-house) Bilingual staff (have 9–10 South Asian bilingual staff)	Face-to-face	3
	Site B (Yorkshire)	Pakistani	Formal interpreters (on-site) Bilingual staff (have 14 South Asian bilingual staff)	Face-to-face	1
Low	Site C (South-east England)	Indian (but interpreting service users usually Bangladeshi)	Formal interpreters	Telephone	1
Moderate	Site D (Greater London)	Indian	Formal interpreters	Face-to-face, telephone	1

Pop., Population.

#### High needs

The primary modality of interpreting at Sites A and B was face-to-face interpreting, with interpreters present on-site regularly due to the impracticalities of arranging per-appointment interpreting for their large non-English speaking population. They used the same interpreters due to the knowledge, rapport, relationships and trust developed with patients over time. Their interpreters lived in the same community as the patients and were able to interact in culturally-sensitive ways. In Site A, this interpreter was employed as a member of practice staff while Site B used locally commissioned interpreters who came for blocks of hours every day.

Respondents in Sites A and B highlighted how beneficial it was to have interpreters on-site and spoke very highly of them, their high-quality work and the difference made to service delivery. They highlighted their: efficiency (able to shorten consultation time by knowing patients, their history and needs); familiarity (serving as connectors and helping to fill gaps); and preferability (patients preferring to book the interpreter when around/available and not minding waiting). They mentioned that these interpreters could be easily *“grabbed”* since on-site and during observations, the researcher could see how interpreters moved seamlessly interpreting for different roles/offices—clinicians, reception staff, waiting area. In Site A, patients often checked whether the interpreter was around rather than the doctor. In Site B, sometimes patients refrained from updating the practice about moving residence to keep accessing their interpreters (*“we’re really popular with people that don’t speak English”*).

**Figure 1:**
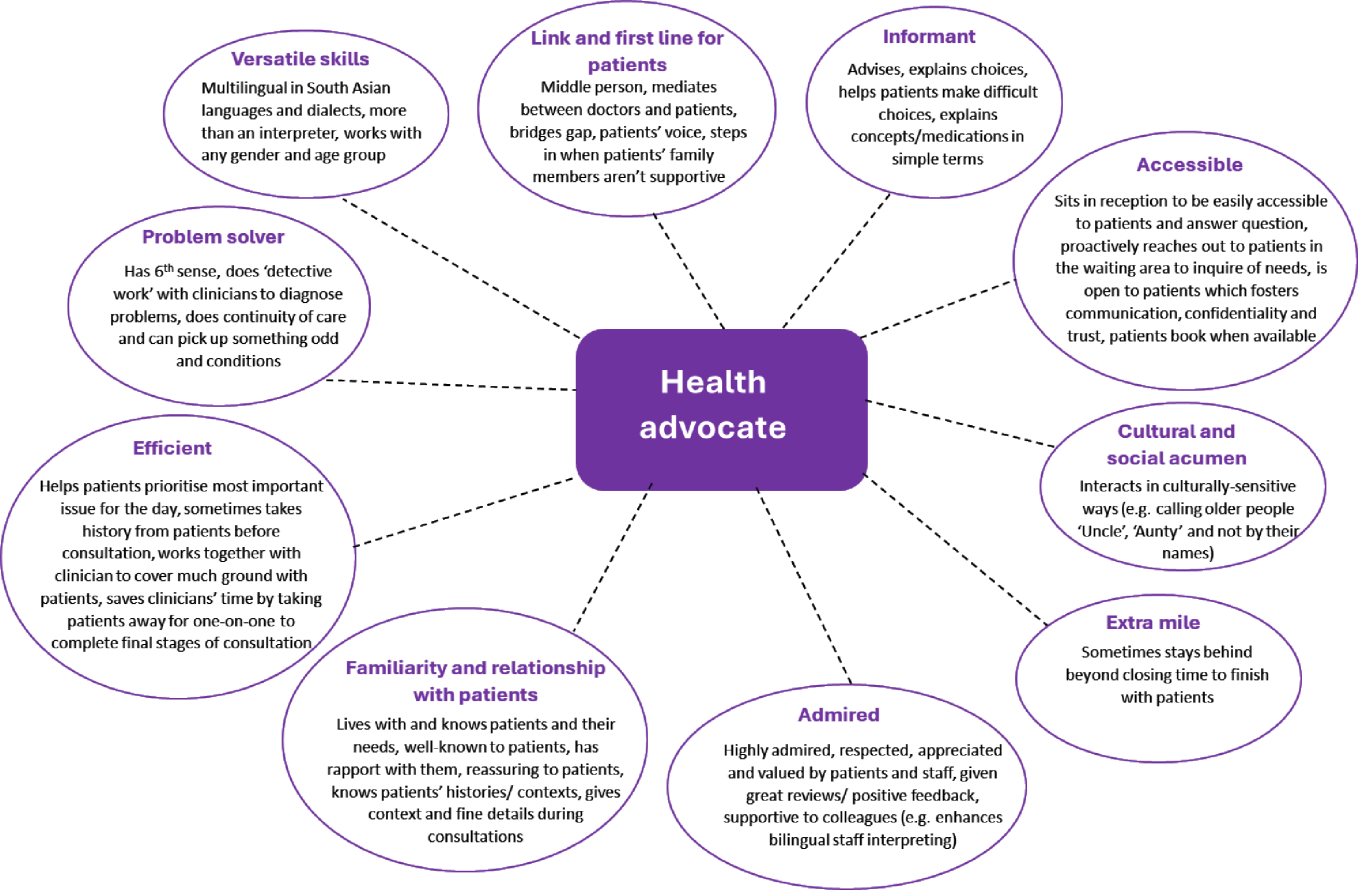
Description of a health advocate by frontline staff.

Site A used the health advocacy model where an interpreter received additional training to provide basic health information to patients and could speak on their behalf in consultations. Practice partners adopted this model as they felt it benefitted their patients, although it cost more and had human resource opportunity costs. Frontline staff described the key roles and impacts that the health advocate had on service delivery and outcomes, including serving as a first line for patients, and having versatile language/dialect skills. The health advocate was seen to have a ‘6^th^ sense’ in picking up subtle changes in patients that then led to diagnoses of serious issues such as cancers and heart disease (figure 1).

In addition to having interpreters on-site regularly, the high needs areas also used other models. Bilingual staff were sometimes used to interpret, with some staff having long work histories at the practice. In Site B, for example, some staff had been working there for 20–30 years and knew the community very well. They started interpreting for their own families as children in the 1970s and 1980s, before being hired as interpreters and then transitioning to further roles. Bilingual staff tended to interpret in circumstantial cases and appeared to confer some advantages of interpreters working on-site. However, a few disadvantages were also reported, such as increased workloads, emotional burden, patients potentially feeling self-conscious/threatened when they saw them on the street where sensitive issues were involved, and dialect differences/language competency issues particularly with respect to second or third generation migrants. Site A staff mentioned that they tried not to use bilingual reception staff as interpreters for some of these reasons, and because they were not trained.

Many frontline staff in high needs areas expressed a strong preference for face-to-face rather than telephone interpreting. They felt that the telephone was inefficient and impractical due to their substantial interpreting needs, and that face-to-face enabled a higher proportion of patients to be seen. Face-to-face interpreting was labelled as “*the gold standard”*, and *”why turn down a five-star service?”* if the health advocate was present. Consequently, telephone interpreting was only used rarely (eg, once a week by a GP) or as a *“last resort”*. In a GP observation session, the researcher saw how visual demonstrations were made by the GP, patient and health advocate using fingers/hands to try and understand the patient’s symptoms/conditions, which may have been difficult with telephone; the GP said it would have been *“impossible”* to have the consultation without the health advocate present.

High needs staff reported that their patients also preferred face-to-face to telephone interpreting due to the trust and relationship built with face-to-face interpreters, the perceived assurance of having their messages interpreted correctly and easier confidence in someone seen rather than *“a person without a face*”/unknown*,* as *“trust is such a massive thing* [in the community]”. Patients also found it difficult to discuss sensitive issues and worried about confidentiality, that is, not knowing if telephone interpreters were local and could share sensitive information with others. Frontline staff noted that patients were a bit more guarded during telephone interpreting as a result and could tell from the tone of some patients’ voices that they were not relaxed and there seemed to be *“a disconnection somewhere”*. Elderly, frail patients also did not like being on the telephone. A few frontline staff mentioned that patients have never requested telephone interpreting; it was a last resort.

Experiences and views about telephone interpreting in high needs areas appeared to be linked to the robustness/reliability of the practices’ respective telephone services. The two high needs sites had somewhat contrasting experiences. Site A staff described their service as *“very good”, “very clear”*, *“brilliant”* and “*have never not been able to get hold of an interpreter”*, although they also reported some challenges. Site B staff spoke almost entirely negatively about their telephone service, a key issue from their interviews. They reported on poor quality including: IT issues (lines dropping, crackly, needing to restart when lines dropped, much time in connecting to the interpreter, long waits that slowed things); varied interpreter quality; and distracted interpreters (eg, hoovering while interpreting). Some staff described the phone service as “*absolutely rubbish”* and “*hit and miss”.* Hence, they tried to avoid it and only used it *“when absolutely desperate”* or when other options were unavailable.

#### Low needs

Site C used telephone interpreting exclusively, as their population need did not warrant an in-house interpreter. The site used telephone triage prior to COVID-19, so the interpreting service integrated easily to their service where the first point of call for all patients (interpreting and non-interpreting users) was telephone. Previously, the site had used a face-to-face service but found that it did not work for their needs due to logistical issues: interpreters needing to travel all over the county; interpreter no-show/unavailability; difficulty scheduling appointments for urgent issues; and the system being unreliable and time-consuming. Additionally, consultations sometimes over-ran and the interpreter had to wait or leave for their next appointment. While the face-to-face option was still available in principle, the site would not use it unless a patient requested it.

In terms of design, the telephone interpreters were randomly allocated and a “*completely different person [is connected] each time”*; they could not request a specific interpreter. Sometimes patients were brought in face-to-face while the interpreter was on the telephone, for example, for conditions that may not lend themselves readily to virtual consultation or for patients whose behaviour was unpredictable.

Several advantages of the telephone interpreting service were highlighted, including: (1) ease of access/equity where patients could be booked same day as everyone else since on-demand; (2) removal of the ‘middle man’ as clinicians could call interpreters directly and not wait for receptionist booking; (3) efficiency, as the service worked well, was more convenient and responsive, gave instant access and was particularly valuable for emergency appointments; (4) having a variety of provision with most languages available and a wider pool of interpreters from across the country; and (5) no need for interpreters to travel or wait for overrunning clinics. Overall, Site C staff sounded satisfied with their on-demand telephone service as it worked well and while variable service was experienced sometimes (box 1), this was largely attributed to patients’ poor phone signal and having ‘off days’.

Box 1 **Sample observation data of telephone interpreting**

**Smooth service**

The interpreted consultation with Patient #2 lasted for 9 minutes (~10 minutes). There were no technical glitches and everything went fine. The Interpreter came on board almost instantaneously after dialling (~4 - 5 seconds afterwards).

**Inconsistent service**

Attempts to set up the 3-way conversation with Patient #1 took ~15 minutes or more, not including the consultation time. It involved multiple interpreters, with a new interpreter coming on board when the process stopped and started. It was at the 4th interpreter that the set-up worked and the consultation took place. Part of this time involved being put on hold (with music playing) and calling an automated line. The interpreter then got cut-off mid-consultation and it was unclear if this was due to connectivity issues, not wanting to interpret the question about a sensitive issue, or distraction/unprofessionality on the part of the interpreter. It was the patient’s wife who completed interpreting for the session. The clinician remarked that this was a frustrating session.

By Patient #3, the clinician had to ask the telephone interpreter at some points if the patient’s son could continue interpreting, as much time had already elapsed due to technical issues that day. The interpreter agreed. The clinician mentioned that they were going to have to reschedule the appointments for the other remaining patients. The clinician wondered if the impacts of the inconsistent service were seen/felt due to an increased demand on the service today, as a clinic with several patients needing interpreters had been scheduled. The clinician remarked that they may not have noticed issues with telephone interpreting previously because they use it infrequently; hence problems encountered in one consultation may have felt ‘quite normal’ since there were fewer impacts.

During another consultation on a different day, the telephone set-up got connected almost instantaneously, however the telephone interpreter also got cut-off eventually and was no longer online mid-consultation. The clinician finished the consultation using the patient’s somewhat limited English proficiency, with the patient’s wife contributing intermittently.

#### Moderate needs

Site D used both face-to-face and telephone interpreting. The face-to-face interpreters were not employed by the practice, but some were regulars and had been interpreting for them a long time. Frontline staff expressed positive views about their telephone service in terms of being quick and straightforward to access an interpreter, covering most languages and being able to use it for smear test. Some staff mentioned challenges such as needing to pre-book certain languages and being unable to do formal physiotherapy examination or other physical procedures; additionally, low audibility was also noticed during an observation session.

The moderate needs site also used bilingual staff, particularly when interpreters did not show up, to support appointment booking or by patient request. Frontline staff reported that patients were appreciative of clinicians who spoke their language and would sometimes specifically request a bilingual clinician rather than an interpreter, as it was seen as cutting out the “*middle-man”*, avoiding being “*lost-in-translation”* and providing a comfortable space to discuss certain topics. Bilingual staff also eased the burden on the practice, as booking interpreters was no longer necessary. However, they also noted difficulty in translating certain words and needing to revert to Google Translate.

### Universal principles across all levels of needs

We found that all sites exhibited similarities in terms of interpreting principles around the use of family members, modality choice decisions, the use of Google Translate and general expectations of service delivery. Older patients tended to rely on their children to deal with their healthcare needs, and family members also filled in the gap when interpreters cancelled bookings or did not show up, or when the practice was particularly busy. While staff reported that some patients felt more comfortable with family members, frontline staff at all sites also noted confidentiality, safeguarding, privacy and quality implications of using family members as interpreters and tended to limit their involvement to non-sensitive consultations or where non-personal information was being given. Frontline staff gave specific examples when they insisted on using professional interpreters despite contrary requests, for example, for a significant family planning decision (table 2). One nurse mentioned that smear tests were never conducted without an interpreter, and considered non-interpreter use for this as abuse of the patient.

**Table 2:** Sample respondent quotes

Objective 1: Delivery of interpreter services	
**Category**	**Sample quote**
Rationale for interpreters	*We realise that a large proportion of our population doesn’t actually feel confident in using English as a first language. So even if they can speak a bit of English sometimes, particularly when describing medical terms or symptoms they might find that difficult you know where as they might be able to get on fine in a local shop or say in you know having a little social with people*. (AF4, clinical staff, Site A)
Models of interpreting delivery by population needs	*High needs* *We have always found that having our own in-house advocate just makes it far more efficient and actually helps us with time management a lot better, rather than sort of waiting in a queue for somebody centralised who may or may not be available at a time when you need them. But also building into the equation the fact that sometimes I might be over running I might have some complicated patients and then the advocate on the other end of the phone needs to be available for a longer period of time.* (AF4, clinical staff, Site A) *Nearly two-thirds of our consultations require an interpreter. So, actually, if you gave a clinician an Urdu-speaking patient and the clinician only speaks English, and you have a clinic full of non-English-speaking patients, waiting for telephone lines to connect and disconnect; we would only probably be able to see about 10 patients. Whereas at the moment, we can see 30 to 40. It makes it easier, so, one of them will take an Urdu interpreter and if it’s urgent on-the-day care, they will keep that interpreter and see all the Urdu-speaking patients and they’ll just keep that one interpreter for the morning. So, the service that we offer, because of our patient population, is absolutely crucial to those interpreters that we have access to face-to-face.* (BF1, administrative staff, Site B) *Low needs* *The new [telephone] system’s so much more convenient and a lot less hassle. Because with the old (face-to-face) system if the patient didn’t turn up then the interpreter’s sat here, sometimes the interpreter didn’t turn up and you were sat with a patient you couldn’t communicate with. So this way it’s much more convenient*. (CF3, clinical staff, Site C) *Moderate needs* *And for most of the patients, if they need an interpreter, it would just be, call the interpreting services and whichever interpreter is available will help out. So that’s mainly for the telephone consultations but sometimes we also need interpreters for face-to-face and at that time that would be pre-booked in by the receptionist*. (DF1, clinical staff, Site D)
Additional quotes on models	*Bilingual staff* *The patients appreciate that and they always want somebody who’s able to speak in their language. You know, I’ve had a patient who has said, “I want a Gujarati-speaking pharmacist,” for example, rather than, “I want a Gujarati interpreter. I would prefer to converse directly with the clinician that speaks the language.*” (DF1, clinical staff, Site D) *Family members* *I mean ideally we shouldn’t use them [family members] because, well there may be a conflict of interest, there may be things that are confidential, and it may just not be appropriate. For example if you’ve got an elderly father and their daughter, and you’ve got testicular problems for example, and there is that cultural barrier in terms of it not being appropriate to talk about those things with your father, for example. So you know, ideally we should be using interpreters, even if you have a family member. But again, time constraints and time pressures, sometimes if the service isn’t running smoothly, you end up just using the family member to translate for you because they’re there. And you presume consent or you can double check with them that, you know, dad’s happy or mum’s happy, but again it comes down the pressures I think that we’re all under at the minute*. (CF1, clinical staff, Site C). *I do fertility clinics and in my fertility clinic where I have a couple, the husband always translates for her normal appointments, but I insisted on there being a translator for where we, I want her to know she is consenting to the treatment and I want her to have me tell her about the treatment and for that to be translated by an external source so that I, 100% knew, that she was happy to go ahead and she understood the process because I don’t know what her husband’s telling her. So sometimes if we feel it’s necessary we will insist upon an interpreter. Neither of them actually wanted an interpreter, but they understood my reasoning for it and so were accepting of it. We have one lady who usually uses an interpreter, but on the screen last week it was to do with mental health and it said, “She has asked not to have an interpreter this time, she wants her husband to speak for her.” So she chooses who she uses*. (CF3, clinical staff, Site C). ***Patients’ preference*** *I have never come across a patient who would prefer telephone interpreting services if a face-to-face was not available. In my experience, telephone interpretation is almost like a choice of last resort because they- … Yes, they want to- and even when you listen to telephone conversations and stuff, the consultation is slightly different, they’re a little bit more guarded*. (BF2, administrative staff, Site B)

Objective 2: Contextual factors impacting interpreting delivery	
**Category**	**Sample quote**
Resourcing and funding formula	*That formula excludes things that are really important, because when it was designed, in 2002, no one thought about language needs, so it’s entirely- And the thing is, they’ve got to base it on national data. So we all collect data on age and sex, and some of the data around deprivation scores are already kind of set across the country. So our formula is (mentions formula), so we get paid- We have money taken off us, at the moment, because nobody counts the fact that 50% of our consultations need an interpreter*. (BF3, clinical staff, Site B)
‘Mop-up’ from secondary care	*When patients go to the hospital appointments and they don’t have an interpreter service, they come here and they say, “Oh, I went to my hospital. What is my result? They said a lot of things. I didn’t understand anything.” … They come and say, “Yeah, I did have an operation but I don’t know what happened.” … And sometimes they don’t know what medication they’re on. They have a bagful of medication when they’re discharged and they don’t know how to take it.*” (AI1, administrative staff, Site A). *Because the access to interpreting services is not very good in secondary care, we often get a lot of work from secondary care pushed to us to explain test results scan results, and future plans for illnesses. Our interpreters will very often be professionally involved with giving results and they're very neutral; so, they don’t make opinions, they interpret what we ask them to interpret*. (BF1, administrative staff, Site B)
Confidence speaking English	*We have realised like a lot of patients don’t feel confident or don’t think they can speak English but yet they can tell us everything in full sentences. …I had a patient … at first she kept asking to speak to someone in Bengali but my colleague was stuck on a very long phone call, but I managed to get all the information…* *She spoke in English and she gave me all the information. But we tend to find that a lot of, it’s not just, it mainly is women that go, “oh I can’t speak English” or you know, but it can be a male patient as well. But sometimes we just have to encourage them to kind of speak in English. If we know they can make out full sentences and tell us what’s going on*. (AF2, administrative staff, Site A)
Interpreter-related and interpreting provider-related issues	*There have been a few occasions when we’ve had to report some telephone interpreters. We had one recently that was actually hoovering whilst she was trying to interpret and all you could hear in the background was her hoovering… Again, they live all over the world, and sometimes, their actual English is not great. So, although they’re translating for us, they often don’t feedback very well, they don’t translate back to us in English very well*. (BF1, administrative staff, Site B)
Lack of interpreter training in medical schools	*What we’re taught at medical school, is when you have an interpreter, the interpreter is supposed to sit there, you’re more or less supposed to ignore them, while it’s a two-way process. Okay? But, like, actually, in reality, that’s not what happens. You speak to the interpreter, and then they speak to them. And I think that that’s what I aim for, when I try and set the phone to speaker, but in reality it doesn’t work like that, and I think there’s no point in really trying to like achieve that model, because it doesn’t work in real life*. (CF2, GP, Site C)

AF2, AF4, AI1, BF1, BF2, BF3, CF1, CF2, CF3, DF1 = pseudonyms/codes for respondents.

**Figure 2:**
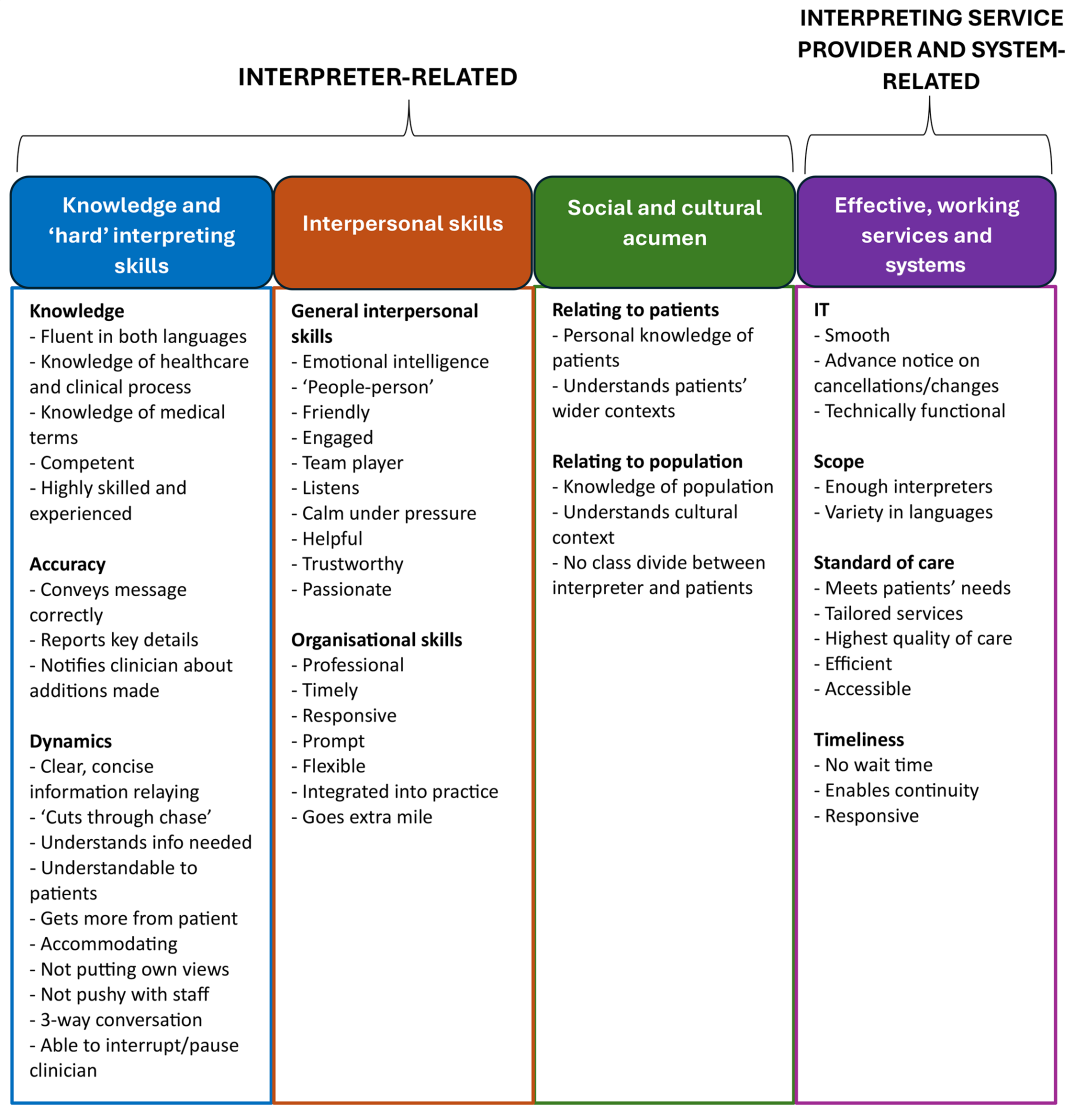
Frontline staff expectations of service delivery. IT, information technology.

Sites with access to both face-to-face and telephone had similar tendencies, using face-to-face for conditions needing physical examination/visual assessment or following patients’ preferences. Patients could request specific named interpreters (with their IDs/codes noted), or interpreters of a particular gender, although clinical need was sometimes prioritised over patient preference. In addition, Google Translate was used as a supporting/complementary interpreting tool across sites, for example: to help bilingual staff translate words that they did not know; for highly literate, non-English speaking patients (eg, recently from abroad); and for staff with non-patient facing roles to have rare/occasional contact with patients. Google Translate was also used in circumstantial situations, for example, when telephone interpreters could not be connected or when a patient presented unexpectedly with a sick child. We also found that video interpreting was rarely used across all sites, except for British Sign Language users. Furthermore, we found that frontline staff across sites had similar expectations of service delivery, which largely focused on interpreters and interpreting service providers. The results suggest that interpreters were expected to have a broad range of skills, and providers were expected to deliver services that work (figure 2).

### Identification of interpreting service users and booking arrangements

We asked case study sites how they identify patients needing interpreters, and how they book their appointments. We found similar approaches across sites, with some site variation. Three primary ways of identification were observed:

1. **Data-based:** This involved noting and recording patients’ language needs/preferred language during GP registration, which then popped up on record systems subsequently. This was common to all sites, with Site B updating the database regularly through an innovative live monitoring system.
2. **Proactive**: Staff made deliberate efforts to make patients aware of interpreting services including poster-hanging in the practice and informing new patients. Staff also identified patients’ need during consultation or at the front desk when patients struggled to communicate or displayed lack of understanding. In addition, patients were identified at key contacts (eg, the 8-week postnatal check), through gentle encouragement to use an interpreter, familiarity and knowledge (eg, certain surnames) and active surveillance (in Site A where the health advocate sat in the reception and reached out to patients).
3. **Passive**: Patients could make requests for interpreters themselves at the front desk/via phone, or have their family members ask on their behalf.

We also asked sites about their interpreter booking processes. All sites had key details for booking appointments, for example, access codes, list of languages, step-by-step guides for initiating calls and help-desk numbers. Only Sites A and B (high need) used interpreters to support patients to book appointments. In Site A, elderly patients were helped particularly with bookings as they struggled with online/e-consult processes, lived on their own or had busy children. Bilingual staff also supported with booking. The low needs site, in contrast, relied on family members to book appointments for patients or used other approaches (getting by with patients’ basic English, non-verbal communication, booking patients pre-emptively if unsure what was needed). Moderate needs areas used a combination of these approaches as well as a translation-enabled query system.

### Contextual factors that impact interpreting delivery

Several contextual factors affected quality and consistency of interpreting delivery (table 2), which are described below.

#### Breadth and complexity of patients’ needs

Patients needing interpreting were generally seen as a group with multiple challenges across need levels. One GP reported that patients were now complex, had comorbidities, complicated tests, missed appointments and ‘jump around [in speech]’ during consultation, adding to the complexity of consultations. Some clinicians also felt that interpreted consultations had intrinsic limitations, for example, clinicians being unable to impart nuances (eg, uncertainty regarding diagnoses/treatment), go in-depth during consultations, nor explore/ask their thoughts, ideas, concerns and expectations. Hence, clinicians felt compelled to maximise consultations when they got good (telephone) interpreters; hence, they overran. They also reported dealing with patients’ non-health/non-medical problems and tasks/issues and extra work that English-speaking practices did not have to deal with, such as supporting patients to go for their hospital appointment, educating patients, translating paperwork about services, and *“a lot of hidden work that NHS doesn’t value”*.

Sites mentioned that some patients were not aware of interpreting services, whereas others requested interpreters despite speaking good conversational English, seen as “*a few questionable requests”*. There were reports that some patients (tended to be men) declined interpreters when they actually had a need and felt offended/*‘a bit insulted’* when offered one. Frontline staff in high needs areas particularly highlighted the importance of encouraging patients to take up basic English classes. They felt that this also had additional benefits such as improving their job prospects and enabling a switch from hard, manual jobs which impacted their health negatively further. Patients who spoke English to a reasonable extent should also be encouraged to speak.

#### Absorbing work from secondary care

Respondents in high need sites mentioned additional work being pushed to primary care and their needing to do ‘mop-up’/follow-up work from secondary care (explaining test results/procedures/medications, future planning for illness and having to deal with fall-out with patients from hospital missed opportunities). They reported that hospitals did not always provide interpreters for patients even when indicated in referral notes, with some hospitals asking GP practices to book interpreters for them. They felt that hospitals were not as competent and confident in using interpreters, unlike GP practices. Staff suggested the need for a centralised interpreting system and improved communication between primary and secondary care, and primary care and other services.

#### Resourcing and the funding formula

Site B staff reported being significantly underfunded. This was seen as driven by the national funding formula which accounts for deprivation but not interpreting needs, nor the earlier onset of ill health in their patient population: “*our 50-year-olds are like other people’s 70-year-olds. And that’s well understood, that in deprived populations, healthy life expectancy is 20 years lower”*. Staff reported that their patients are having heart attacks, strokes and diabetes in their 30s, ahead of the average.

Site B mentioned that *“our days are much more difficult”*, “*every little piece of work takes longer”* and that interpreted consultations took longer, with double appointments being impractical if a substantial proportion of the patient population needs interpreting. In addition, they felt that they needed more staff, as “*if 50% of our conversations need an interpreter, we need 50% more staff”.* They reported losing some GPs due to workload, which was “*getting heavier and heavier”*, and that while their staff could work in other affluent areas with less stress, they had opted to stay back to make a difference. They mentioned highlighting these challenges to policymakers extensively, yet reported recognition of these problems with not much being done as the system was overwhelmed across the board and being “*ignored”* and left to “*get on with it”*, which they attributed to institutional racism. Site A did not experience these challenges, however. Staff acknowledged that while interpreted consultations took time, some consultations could be completed within 10 minutes; some patients would need less time than others and vice versa, with some balance overall. This assertion was also acknowledged by a few Site B staff. High needs sites felt they would benefit from more face-to-face interpreters as staff, hiring more bilingual staff and getting interpreters that understood patients’ wider socio-economic contexts and those who had worked in other public sectors.

#### Frontline staff interpreting training and attitudes

Some staff were concerned about a lack of training in medical schools and on-the-job training on using interpreters, including being taught in medical school to *“ignore”* the interpreter and have a two-way conversation with the patient, which did not work in reality (table 2). A few suggestions were made on relating to interpreting service users. Frontline staff were encouraged to have the right attitude and be patient when speaking with patients through an interpreter: being flexible; speaking slowly, clearly and giving patients time to ask questions; not panicking or assuming that an interpreted consultation would be difficult; and doing full blood count when symptoms were nebulous and a diagnosis could not be deciphered. Staff interpersonal skills and social prescribing in Site A were seen as enablers of service delivery. Some frontline staff created unique solutions that facilitated their own consultations. For example, during telephone consultations, a moderate needs staff member allowed interpreters to introduce themselves/speak first to the patient rather than the health professional, as historically, patients would automatically pass on the phone to a family member once they heard English at the other end or shout into the phone.

#### Interpreter-related and interpreting provider-related factors

Delivery was also affected by interpreter factors, for example, no-shows, late arrivals, being distracted/unengaged and rushing and not staying to help patients with follow-on tasks such as booking follow-up appointments (table 2). Some interpreting providers seemed to employ less skilled interpreters with reports of poor spoken English, not interpreting accurately and dialect differences. Low pay, poor working conditions and unfavourable terms and conditions given to interpreters were also seen as impacting delivery quality. A few respondents highlighted the need for recognition of interpreting skills in the system/country and creating opportunities for new interpreters to come on board. For instance, a high needs staff member suggested harnessing the skills of young people who were already fluent in multiple languages but currently in low-paid, back-breaking jobs and who were feeling disillusioned/marginalised; this step was seen as able to boost their confidence/morale, increase aspirations and break the cycle of generational sickness.

There were also system factors relating to interpreting service providers (eg, IT issues, codes not working properly, lines dropping and no prior notifications of cancellations). Some sites were proactive in switching from services that did not work for them to new ones that met their needs. Site C reported the benefits of scheduling patients who need an interpreter at the same clinic, which came as a direct result of their participation in our study. Particularly, this helped with continuity of care, especially when patients saw the same clinician.

## Discussion

This is the first study to our knowledge to report on the delivery and implementation of interpreting services in UK primary care. A particular novelty of our study is the use of contrasting case study sites in areas of high, moderate and low interpreting need, and the focus on long-term, multigenerational residents instead of refugees or asylum seekers on which existing evidence has predominantly focused.

### Main findings

We found that primary care practices typically organised and delivered interpreting services according to their local population needs, including different models and modalities of interpreting. High needs practices with high migrant populations predominantly used face-to-face interpreting with interpreters on-site regularly, which was perceived to have manifold benefits. In contrast, low needs areas primarily used telephone interpreting due to perceived ease of access, widened scope of languages and overcoming interpreter travel constraints. All case study sites made some circumstantial use of Google Translate and family members for interpreting. There was very little reported use of video interpreting. Quality and consistency of delivery were affected by local healthcare pressures, particularly from secondary care. Patients requiring interpreters were perceived to have more complex healthcare as well as language needs, which were seen as not reflected in financial resourcing. Delivery was also challenged by interpreting service providers’ systems and varied quality of interpreters.

### Interpretation and implications

The CFIR has been widely used to examine healthcare implementation,^28,29^ including some efforts to address inequalities in healthcare delivery. Our study demonstrated that interpreting is a complex intervention with both practical, cost, training and ethical considerations; several modalities and models can be adopted for different contexts and population needs. We found that primary care sites delivered interpreting services according to their local population needs. This is consistent with standard commissioning guidance where primary care contexts are able to provide services tailored to their populations.^34^ High needs practices favoured on-site, face-to-face interpreting due to perceived benefits for both patient care and healthcare management. However, financial resourcing for interpreting services did not adequately reflect the increased complexity of care for patients with limited English proficiency, further exacerbating disparities in service delivery. While toolkits and guidelines have been proposed to address such challenges,^35^ limited resources may challenge their use. As the inverse care law shows,^36,37^ people with the greatest health needs often have the poorest access to healthcare. Language barriers contribute to health inequities by impeding communication in healthcare, thereby limiting access to good quality care.^2,4,14^ Providing interpreting services could help mitigate these health inequities.

Our case study approach illuminated the differences in inner settings and how these affected delivery. For example, there were marked differences in the quality of interpreters, which directly impacted the ability of practices to deliver consistent and effective interpreting. Respondents across all sites had similar expectations of interpreter skills and interpreting delivery systems, which suggests that frontline staff want services that work and enable them to perform their duties. The outer setting was a dominant factor affecting delivery, with resourcing affecting practical aspects of interpreting as well as attitudes and values. Both high needs sites used predominantly face-to-face interpreting, and one site was willing to use the more costly Health Advocate model to reduce the burden on staff. This aligns with evidence suggesting that professional interpreters enhance care quality, particularly in contexts requiring complex communication.^38^

Our study has shown that the choice of interpreting modality is largely affected by local context and commissioning. This may have implications for patient satisfaction and uptake. Although a systematic review of patient satisfaction with telephone or video interpreting compared with face-to-face reached no conclusions about patients’ perceptions of superiority of modality,^39^ there is evidence that healthcare providers and interpreters prefer face-to-face interpreting.^40^ Healthcare interpreters perceive telephone interpreting to have more negative impacts than video interpreting on their interpreting performance, their rapport with the interlocutors, concentration, stress and fatigue.^41^

Our findings highlight persistent issues with access to professional interpreters in primary care settings, which arise despite their critical role in facilitating effective communication with patients with limited English proficiency.^35,42^ All sites used family members and, in some cases, Google Translate as ad hoc solutions, despite the availability of professional interpreting services. This reinforces concerns raised by Cramer,^38^ who argued that reliance on ad hoc interpreters or no interpreters undermines the quality of care for patients. Previous studies have also highlighted risks of Google Translate as a translation tool, including accuracy issues.^17,18,20,43^

### Strengths and weaknesses

By adopting a multisite case study approach with diverse primary care practices and 22 interviews, we were able to capture how different populations influence delivery. We did this across a wide range of geographical and demographic settings. This makes our findings highly generalisable to different areas of the country and variation in provision. Our emphasis on local needs has produced a rich account of everyday practice as well as targets for improvement. By grounding our data collection and analysis in the CFIR, we have aligned our study with other implementation research and strengthened its relevance to the wider field of healthcare improvement.

Our study is limited by the focus on South Asian populations in the UK, which may have some implications for generalisability to other ethnic groups, although previous evidence elsewhere suggests that different patient ethnic groups and frontline staff share similar expectations of interpreting service delivery.^44–46^Cultural factors could also affect the need and experiences of interpreting, for instance, the preference for family members as interpreters in some communities. Some of our findings report on staff perceptions of patient experiences of interpreting, and while our broader INTERPRET-X study included patient perspectives,^14,47^ we acknowledge some possible differences in viewpoints.

Furthermore, we do not know how provision differs in areas with a greater diversity (rather than density) of languages spoken. It is plausible that certain modalities favoured in high needs areas may be difficult to implement in settings with greater diversity of languages spoken due to logistics and resource constraints of having multiple, regular, on-site face-to-face interpreters. We also acknowledge the varied history of migration in England and how this affects local communities and healthcare practices. Lastly, our study is limited by a lack of data on wider social determinants of health, such as how economic stability, education and social capital affect interpreting provision.

### Recommendations and future research

This study highlights several actionable recommendations for improving interpreting service delivery and reducing health inequalities, alongside key areas for future research.

This study underscores the need for primary care providers to have a wide range of options to tailor delivery to their population needs, and supported with the needed resources for delivery. The quality of interpreters and third-party interpreting providers are significant determinants of service effectiveness. Interpreters working in primary care should have satisfactory qualifications, skills and training, but this should also be backed by better pay and working conditions to improve their service delivery.^48^ The NHS should strengthen procurement by engaging in meaningful dialogue with interpreting providers and collecting robust data on service performance. Future research should explore the cost-effectiveness of investing in different models of high-quality interpreting services, the emergence of digital models of interpreting and examining what provision modalities yield long-term benefits.

Bilingual healthcare professionals play a vital role in bridging language barriers, particularly in areas of high need, yet there is a need to validate and assess their levels of proficiency systematically. Future research should focus on developing robust frameworks for evaluating bilingual staff competencies, alongside exploring potential unintended consequences of their use. Healthcare professionals, both pre-service and in-service, should be given appropriate training on using interpreters, which must reflect real-life scenarios.

Finally, further research should examine other integrated contexts and environments for interpreting provision, including unscheduled care and community clinics. Other most commonly spoken languages in the UK, such as some European and Middle Eastern languages, should also be included.^1^

## Conclusions

Our study has demonstrated significant variation in the delivery of interpreting services across four case study sites in primary care, driven by differing population needs, systemic pressures and resource allocation. Practices serving high language needs populations demonstrated the benefits of face-to-face interpreting with interpreters on-site regularly, while those in low needs areas often relied on telephone interpreting. Gaps in service quality and reliance on ad hoc solutions, such as family members or Google Translate, underline persistent challenges. NHS guidance, procurement and funding models should support delivery of tailored, patient-centred approaches in local contexts and facilitate systemic reforms to improve quality of interpreting services.

## Supplementary material

10.1136/bmjph-2025-003454 online supplemental file 1

## Data Availability

Data are available upon reasonable request.
