# Supplementary material for: Delivery of interpreting services in UK primary care by population needs: a multisite case study
Source: BMJ Public Health. 2026 Mar 31;4(1):e003454. doi: 10.1136/bmjph-2025-003454 (PMC13052783; doi:10.1136/bmjph-2025-003454)
Supplement: online supplemental file 1 [file bmjph-4-1-s001.pdf]

Supplementary materials

Table of Contents

Supplementary file 1: Overview of case study sites 2

Supplementary file 2: Frontline staff interview questions 3

Supplementary file 3: RREAL sheet for INTERPRET-X study (Name of site)........ 5

Supplementary file 4: Overview of interview respondents across all case study sites (n=22)........ 7

Supplementary file 5: Overview of observations (total number of episodes/spaces= 24) 8

Supplementary file 1: Overview of case study sites

Site #	England region	Residence	IMD score for practice area*	Main South Asian ethnicity in practice	Practice size range (# of patients)
Site A	Greater London	Urban	3	Bangladeshi	5,000- 7,000
Site B	Yorkshire	Urban	1	Pakistani	8,000- 10,000
Site C	South-east	Rural	6	Indian (but interpreting service users usually Bangladeshis)	5,000- 7,000
Site D	Greater London	Urban	4	Indian	25,000- 30,000

* IMD= Index of Multiple Deprivation

Supplementary file 2: Frontline staff interview questions

Staff role and overview of experience using interpreter services

1. I was wondering if we could begin with a description of your current role.

2. Could you give me an overview, in broad terms, of your experiences arranging and using interpreters with patients whose first language is not English?

Arranging interpreters

I am going to ask a few questions about how you arrange for interpreters.

1. How do you initially communicate with patients whose first language is not English?

2. How do you know whether or not a patient might need an interpreter?
    - *Prompt*: Any indication of this information on the patient's record?

3. How do you offer interpreters to patients? What is the process for accessing and arranging interpreters?
    - *Prompt*: Are patients able to choose the mode of interpreting they use (i.e., face-to-face, telephone or video)
    - *Prompt*: Can specific interpreters be requested for (e.g., the same interpreter for continuity of care, or an interpreter of the preferred gender)?

Perceptions of interpreting services

Now, I am going to ask a few questions about your views on interpreter services.

1. What do you consider a high quality interpreter service (e.g, features etc)?
    - *Prompt*: How does this compare to the services you use?

2. What's your assessment of how well patients understand the information (diagnosis/treatment options) interpreted to them regarding their health?

Patients' needs and experiences

1. Are there times where patients may require other types of interpreters as well as for language? For example, sign language for those who are deaf or hard of hearing?

2. Can you talk about times when a patient has arrived with nobody/no interpreter? How does it affect the consultation?

Barriers and facilitators to using interpreter services

We are now getting to the last sets of questions.

1. What would you say are the challenges for patients to accessing and using interpreters? What are the issues or difficulties regarding access to interpreter services from your practice's perspective?
    - How does the patient's condition/urgency affect this?
    - *Prompt*: Patient reluctance/difficult to arrange/too long for appointments

2. What reasons are given where patients may be reluctant or turn down interpreter services?

3. What do you think would encourage patients to use interpreter services? What facilitates the access and use of interpreters for patients?

4. What do you think would make it easier for healthcare professionals/frontline staff to access and use interpreter services? What facilitates the access and use of interpreters for healthcare professionals and practices?

Closing

- Ask if there's any other thing participant wants to say on the topic
- Thank participant and close
- Turn off audio-recorder

Supplementary file 3: RREAL sheet for INTERPRET-X study (Name of site)

**Context**	Site	
	Size (patient pop.)	
	IMD score	
	Total # of frontline staff	
	Total # of GPs	
	# of years in operation since incep.	
	Stand-alone or part of group	
	Main South Asian ethnicity	
	Proportion of each SA ethnicity	
	Length of interpreting service use	
	Data sources	
	Codes	
	Key information	
**Intervention characteristics**	Arranging interpreters	
	Model of interpreting	
	Modality of interpreting	
	Interpreting processes	
**Inner setting**	Interpreting policies	
	Culture of practice	
	Resources	

Outer setting	Commissioning of interpreting services	
	Local pressures/issues	
	National policies	
Characteristics of individuals	General perceptions of interpreter services	
	Patients' needs and experiences	
	Training on interpreting/ health advocacy	
Processes	Barriers	
	Enablers	
	Any other comments	

Supplementary file 4: Overview of interview respondents across all case study sites (n=22)

Descriptor	Frequency (%)
**Role**	
GP	6 (27.3)
Nurse	2 (9.1)
Other clinicians (HCA, physio, clinical pharmacist, PA)	4 (18.2)
Reception staff	5 (22.7)
Admin. staff	4 (18.2)
Health advocate	1 (4.5)
**Gender**	
Male	6 (27.3)
Female	16 (72.7)
**Ethnicity**	
Asian or British Asian	10 (45.5)
Black or Black British	2 (9.1)
White British or White Other	9 (40.9)
Mixed	1 (4.5)
**Number of years of experience**	
<10 years	6 (27.3)
10-19 years	7 (31.8)
≥20 years	5 (22.7)
Unclear/missing	4 (18.2)
**Number of years in current role**	
<10 years	16 (72.7)
10-19 years	0 (0.0)
≥20 years	4 (18.2)
Unclear/missing	2 (9.1)

Supplementary file 5: Overview of observations (total number of episodes/spaces= 24)

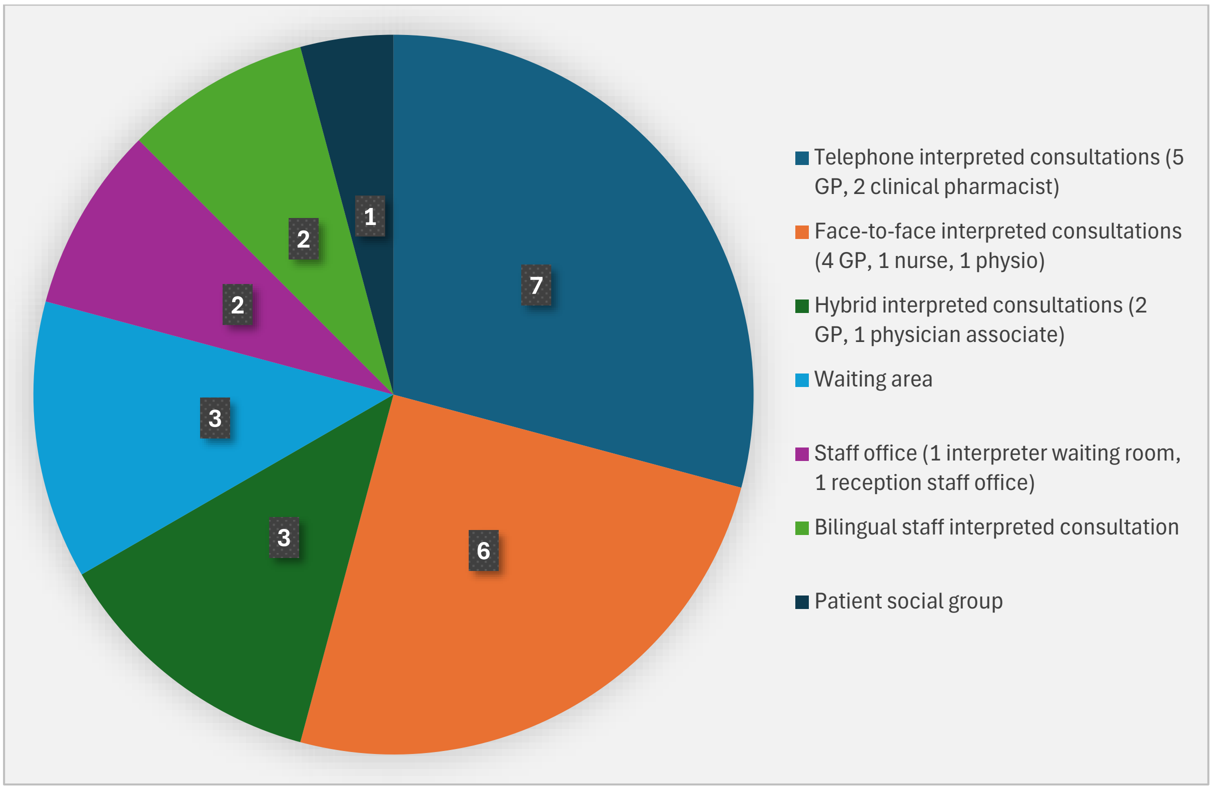

* Patients were not gotten on phone in two of the telephone interpreted consultations.
